# Intuitionistic Fuzzy Biofeedback Control of Implanted Dual-Sensor Cardiac Pacemakers

**DOI:** 10.3390/bioengineering11070691

**Published:** 2024-07-08

**Authors:** Hussain Alshahrani, Amnah Alshahrani, Mohamed Esmail Karar, Ebrahim A. Ramadan

**Affiliations:** 1 Department of Computer Science, College of Computing and Information Technology, Shaqra University, Shaqra 11961, Saudi Arabia; halshahrani@su.edu.sa; 2 Department of Computer Science, Applied College, Princess Nourah bint Abdulrahman University, Riyadh 11671, Saudi Arabia; aaalshahrani@pnu.edu.sa; 3 Department of Industrial Electronics and Control Engineering, Faculty of Electronic Engineering (FEE), Menoufia University, Menouf 32952, Egypt

**Keywords:** cardiac diseases, dual-sensor pacemakers, intelligent control, intuitionistic fuzzy logic

## Abstract

Cardiac pacemakers are used for handling bradycardia, which is a cardiac rhythm of usually less than 60 beats per minute. Therapeutic dual-sensor pacemakers aim to preserve or restore the normal electromechanical activity of the cardiac muscle. In this article, a novel intelligent controller has been developed for implanted dual-sensor cardiac pacemakers. The developed controller is mainly based on intuitionistic fuzzy logic (IFL). The main advantage of the developed IFL controller is its ability to merge the qualitative expert knowledge of cardiologists in the proposed design of controlled pacemakers. Additionally, the implication of non-membership functions with the uncertainty term plays a key role in the developed fuzzy controller for improving the performance of a cardiac pacemaker over other fuzzy control schemes in previous studies. Moreover, the proposed pacemaker control system is efficient for managing all health-status conditions and constraints during the different daily activities of cardiac patients. Consequently, the healthcare of patients with implanted dual-sensor pacemakers can be efficiently improved intuitively.

## 1. Introduction

The sinoatrial (SA) is the biological heart pacemaker and is responsible for generating electrical signals that cause contraction of the atria. It passes through the atrioventricular node, together with the heart conduction system and His-Purkinje fibers, to the ventricles [1]. Thus, a normal heartbeat rhythm depends on the normal activity of the sinoatrial node, and any failure in these systems may lead to heart abnormalities, such as bradycardia diseases [2]. Heart rhythm abnormalities and heart failure patients are mainly treated using pacemakers and defibrillators [3]. In 2023, it is expected that 1 to 1.4 million pacemakers will be implanted worldwide [4,5].

**Figure 1:**
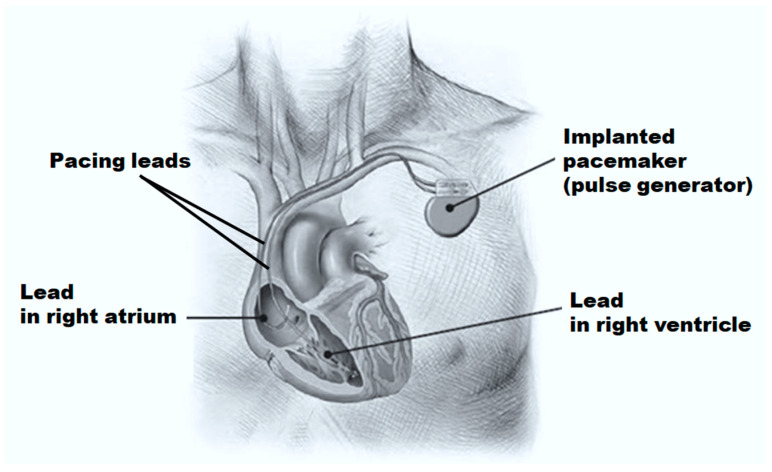
Implanted pacemaker with pacing leads connected to the heart.

Cardiac pacemakers are implanted as depicted in Figure 1. They are used for handling bradycardia, which is a cardiac rhythm usually less than 60 beats/min. The pacemakers aim to restore the normal electromechanical activity of the heart by generating electrical pulses [6]. Sending an electrical pulse to both ventricles to aid them in beating in a more coordinated and synchronized mode recovers the normal ventricular contractility and improves the pumping efficiency of the heart [7]. The main components of traditional pacemakers are pacing leads and a pulse generator [2]. The pulse generator includes the battery of the pacemaker in addition to the circuits that transfer stimulation to the heart only in case of arrhythmia. Pacemaker leads are electrical conductors covered by insulators. The electrical impulses are carried by the leads from the pacemaker to the heart and the sense amplifiers signal from the heart to the pacemaker [7].

The technology of cardiac pacemakers has rapidly advanced since the first pacemaker implantation in the 1950s [8], including the quality of pacemaker leads, monitoring methods, control algorithms, and responsive programming for pacing rates. Different pacemaker types can be classified depending on many viewpoints. Pacemakers can be with or without leads, which are very small devices to be placed within the heart to avoid the use of pacing leads. Pacemakers have internal batteries or can harvest energy remotely by using an external battery pack. 

Heart diseases are some of the most widespread diseases globally. Therefore, implantable cardiac devices are needed to monitor and control heart rates, communicating with medical experts to achieve some therapeutic procedures if the behavior of a patient’s heart becomes abnormal in real time [9,10,11]. Hence, the development of advanced pacemaker control has recently acquired significant attention from researchers. The suggested approaches for controlling the artificial pacemaker have concentrated mainly on using classical linear system theory [12,13], analog spiking neural network [14], and radial basis function (RBF) neural network [15], and different schemes of optimal controllers have been proposed [16,17]. However, the above controllers are still limited to accurate and linearized models of the heart.

Fuzzy logic has been applied in medical applications and diagnosis [18] to overcome complex modeling and parameter uncertainty. The basic idea of fuzzy logic control (FLC) is to qualitatively merge the knowledge of expert physicians into the design procedure of automated medical systems [19,20]. However, traditional fuzzy controllers cannot always give good control actions with respect to the degree of non-membership of all elements in fuzzy sets to manage the system uncertainty [21]. Hence, Atanassov [22] proposed the intuitionistic fuzzy set (IFS) to provide a new general fuzzy framework, comprising both membership and non-membership function degrees at the same time. The IFS has been utilized in medical applications and decision making successfully [23,24]. 

In this article, we aim to propose a new IFL control to regulate implanted dual-sensor pacemakers under different daily activities. The following summarizes the contributions of this study:

- Proposing a novel IFLC of implanted pacemakers to regulate heart rates during the daily activities, i.e., at rest, walking, and jogging, of cardiac patients;
- Investigating the robust capabilities of our proposed IFLC to verify the safe health conditions of patients with pacemakers during daily activities;
- Conducting a comparative study among the current state-of-the-art control methods of dual-sensor pacemakers to validate the outperformance of the proposed IFLC.

The technological advancements of pacemakers have evolved from steady rate to complicated rate-responsive pacemakers in the last few years. Implanted pacemakers sense heart arrhythmias and transmit electrical pulses to stimulate the cardiac muscle and control the performance of the heart pacing [25]. Automated control algorithms have been proposed to improve the efficiency of pacemakers in previous studies. The performance of implanted pacemakers can be classified according to the control approaches and the system variables as open-loop [26] or closed-loop [27,28,29] controllers. For instance, Arunachalam et al. [9] presented a fractional-order proportional–integral–derivative FOPID control of pacemakers to provide the required control signals to organize the cardiac pacing rhythms. Although the proposed FOPID controller is suitable for rate-adaptive pacing, its design is essentially based on selective tuning processes. Also, a proportional–integral–derivative (PID) scheme was proposed to control the heart rate of a pacemaker [30]. It is represented through a loop consisting of the Massachusetts Institute of Technology (MIT) rule with a delta rule and an adaptive correction factor. This proposed PID controller achieved a good transient response in a simulation study, but the adaptive correction factor of the learning rate must be adjusted manually to achieve the stabilized pacing performance. A backstepping control scheme was developed to improve the pacemakers’ performance in managing heart rates by using RBF networks [15]. The RBF network controller has been confirmed by using 12 cases in four arrhythmias patients. However, the RBF-based backstepping controller is complex and should be manually tuned to achieve targeted pacing rates. 

Nawikavatan et al. [16] presented a metaheuristic optimization algorithm to enhance the performance of proportional–integral–derivative–accelerated (PIDA) for controlling the cardiac pacemaker in the cardiovascular system. The spiritual search has been used for tuning both the parameters of the traditional PID and the proposed PIDA control schemes. The results showed that the dynamic response of PIDA control is better than that of a conventional PID for the heart-rate regulation of the pacemaker.

Wojtasik et al. [31] described a brief work of fuzzy logic controller algorithms on hardware implementation for adaptive heart rates. This controller was executed on a CPU and mixed-mode VLSI chip to verify that the pacemaker can be implemented with minimal power consumption. Nevertheless, the main drawback is the comparatively high-power consumption using a commercial CPU core. PID combined with traditional fuzzy control is utilized for designing a control algorithm for the dual-sensor pacemakers [32]. For patients with bradycardias at rest, the suggested fuzzy PID control of pacemakers is utilized. The simulation results showed that this fuzzy PID control is efficient for heart-rhythm recovery, but it is still limited for other body activities, such as jogging cases. Recently, the authors in [33] proposed a robust fractional-order PID (PI^λ^D^δ^) controller dependent upon a particle-swarm optimization (PSO) algorithm for a pacemaker to control the arrhythmias of cardiac patients. Also, Nako et al. [34] used a fractional-order PID (PI^λ^D^μ^) controller to achieve a minimum active component count design to apply in cardiac pacemakers based on simulation experiments. Nevertheless, our study focuses on the development of a novel controller of implanted dual-sensor pacemakers to handle the uncertainty of all possible body activities, such as walking and jogging.

## 2. Methods

### 2.1. Mathematical Pacemaker Model

**Figure 2:**
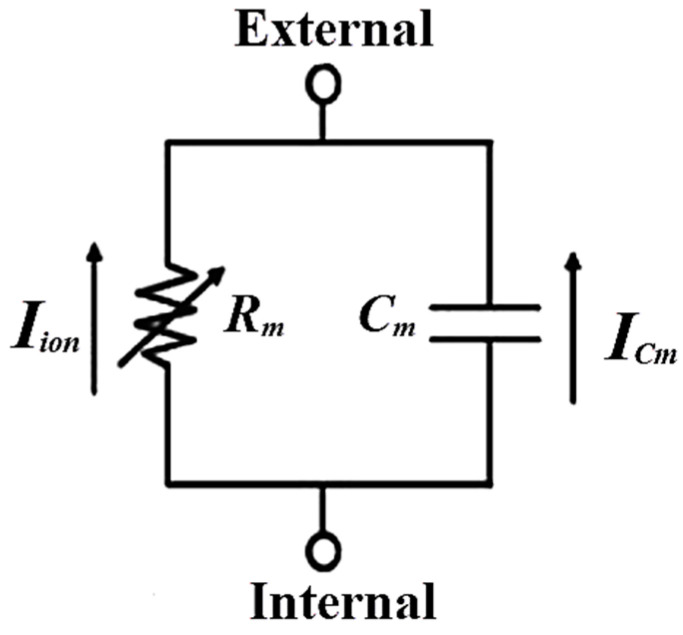
Equivalent circuit model of the myocardial membrane.

This study used the mathematical SA model by Yanagihara, Noma, and Irisawa (YNI) [35]. The YNI model is a good SA model for representing the generation and propagation of the electrical action potentials in the cardiac muscle. It is used efficiently in the numerical simulation of heart electroactivity of SA cells, which are more physiologically relevant models than other mathematical models. In Figure 2, the YNI model simulates the biopotential activity of the heart. This model includes one time-independent current and five dynamic currents. The first is the cell membrane capacitance current *I_c_*, the sodium current *I_Na_*, the slow inner current *I_s_*, the potassium current *I_K_*, and the delayed inner current activated by hyperpolarization *I_h_*, ensuring the conservation of transmembrane currents, in units of (μA/cm^2^). The second is the time-independent one, which is leakage current *I_l_*. The YNI model output response is *V* (mV), which illustrates the membrane potential is given in (1); where *C_m_* (μF/cm^2^) presents the myocardial membrane capacitance and *I_app_* is the applied external current.

(1) $$I_{app}=C_{m}\frac{dV}{dt}+I_{Na}+I_{K}+I_{l}+I_{s}+I_{h}$$

Then, the SA model can be simplified to

(2) $$C_{m}\frac{dV}{dt}+\frac{V}{R_{m}}=I_{app}$$

where *R_m_* presents the myocardial membrane resistance. For the pacemaker, the pulse current *I_pulse_ (t)* is equal to *I_app_* which passes through the resistance *R_pulse_* to produce the voltage *V_pulse_*, and the total transferred energy *E_T_* can be calculated for a single pulse through the duration *d_pulse_* by

(3) $$E_{T}=\frac{d_{pulse}V_{pulse}^{2}}{R_{pulse}}$$

The correlated transferred energy *E_T_* of the implanted pacemaker and the optimal signal pulse have been estimated previously in [36], such that *R_m_* = 20 Ω, *R_pulse_* = 601 Ω, and *V_pulse_* = 197.8 mV. The delay time duration is 0.094 s for recovering each heartbeat. In this study, the pulse duration *d_pulse_* represents the control signal to the cardiac pacemaker to obtain the targeted heart rates during different body activities.

### 2.2. Intuitionistic Fuzzy Sets

Atanassov [37] proposed an intuitionistic fuzzy set (IFS) to generalize traditional fuzzy sets by adding a hesitancy or uncertainty element, linking the fuzzy membership function with the opposite non-membership function. This section gives some basics and concepts of IFS to explain the contributions of our developed pacemaker control system as follows. Let $A^{*}\subset Y$ be a crisp and fixed set. An IFS *A* in *Y* is determined by

(4) $$A=\left\{\langle x,\delta_{A}\left(x\right),\;\beta_{A}(x)\rangle |x\in Y\right\}$$

which is characterized by a membership degree $\delta_{A}\left(x\right)$ and non-membership degree $\beta_{A}(x)$ where$\;\delta_{A}\left(x\right):Y\to \left[0,1\right]$ $\beta_{A}\left(x\right):Y\to \left[0,1\right]$, such that

(5) $$0\leq \delta_{A}\left(x\right)+\beta_{A}\left(x\right)\leq 1\;\forall x\in Y$$

The uncertainty or hesitancy of the element x∈Y to the IFS *A* is given in (7), where $\sigma_{A}\left(x\right)$ is the degree of uncertainty of *x* ∈ *Y*. The IFS *A* is reduced to a traditional fuzzy set if $\sigma_{A}\left(x\right)=0$, ∀x∈Y.

(6) $$\sigma_{A}\left(x\right)=1-\delta_{A}\left(x\right)-\beta_{A}\left(x\right)\;,\;\forall x\in Y$$

Let $a=\left(\delta_{a},\;\beta_{a}\right),\;a_{1}=\left(\delta_{a_{1}},\;\beta_{a_{1}}\right),\;{and\;a}_{2}=\left(\delta_{a_{2}},\;\beta_{a_{2}}\right)\;$be three intuitionistic fuzzy numbers (IFNs) to determine the following four operational rules:

(7) $$a_{1}\;⨂\;a_{2}=\left(\delta_{a_{1}}\delta_{a_{1}},\beta_{a_{1}}+\beta_{a_{2}}-\beta_{a_{1}}\beta_{a_{2}}\right)$$

(8) $$a^{\gamma }=\left(\delta_{a}^{\gamma },\;1-{\left(1-\beta_{a}\right)}^{\gamma }\right)$$

(9) $$na=\left(1-{\left(1-\delta_{a}\right)}^{n},\;\beta_{a}^{n}\right)$$

(10) $$a_{1}+a_{2}=\left(\delta_{a_{1}}+\delta_{a_{2}}-\delta_{a_{1}}\delta_{a_{2}},\beta_{a_{1}}\beta_{a_{2}}\;\right)$$

Let $a=\left(\delta_{a},\;\beta_{a}\right)\;and\;b=\left(\delta_{b},\;\beta_{b}\right)\;$be two intuitionistic fuzzy suggestions and $\delta_{a},\;\beta_{a},\delta_{b},\;\beta_{b}\in Q$ holding the constraints: $\delta_{a}+\beta_{a}\leq 1$ and $\delta_{b}+\beta_{b}\leq 1$. Then, the suggestions *a* and *b* processes, conjunction (⋀), disjunction (∨), implication (→) and standard negation (¬), are given by

(11) $$a\;⋀b=\left(\min\left(\delta_{a},\delta_{b}\right),\min\left(\beta_{a},\beta_{b}\right)\right)$$

(12) $$a\;⋁b=\left(\max\left(\delta_{a},\delta_{b}\right),\max\left(\beta_{a},\beta_{b}\right)\right)$$

(13) $$a\;\to b=\left(\max\left(\beta_{a},\delta_{b}\right),\max\left(\delta_{a},\beta_{b}\right)\right)$$

(14) $$\neg \;b=\left(\beta_{a},\delta_{a}\right)$$

**Figure 3:**
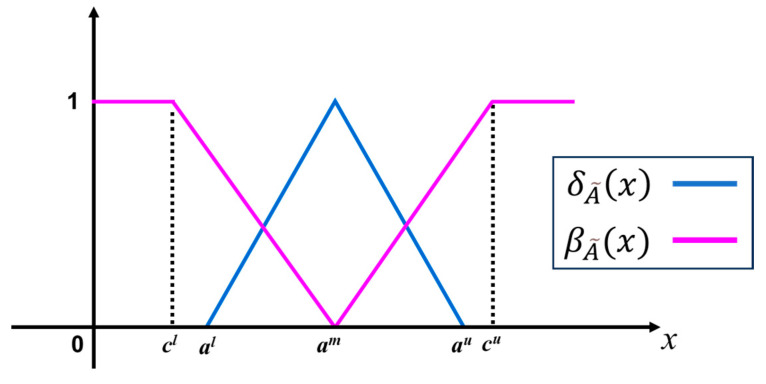
A triangular intuitionistic fuzzy set includes the membership and the non-membership functions.

The graphical representation of a triangular intuitionistic fuzzy set is shown in Figure 3. Assuming the IFN $\tilde{A}=\left(a^{l},a^{m},a^{u};c^{l},a^{m},c^{u}\right)$ such that *c^l^* ≤ *a^l^* ≤ *a^m^* ≤ *a^u^* ≤ *c^u^* and *c^l^*, *a^l^*, *a^m^*, *a^u^*, *c^u^*∈ℝ. The mathematical description of membership function $\delta_{\tilde{A}}(x)$ and non-membership function $\beta_{\tilde{A}}(x)$ are defined as

(15) $$\delta_{\tilde{A}}(x)=\left\{ \begin{array}{l} 0,\;x\leq 0\\ \frac{x-a^{l}}{a^{m}-a^{l}},\;a^{l}<x\leq a^{m}\\ \frac{a^{u}-x}{a^{u}-a^{m}},\;a^{m}\leq x<a^{u}\\ 0,\;x\geq a^{u} \end{array}\right.$$

(16) $$\beta_{\tilde{A}}(x)=\left\{ \begin{array}{l} 1,\;x\leq c^{l}\\ \frac{x-a^{m}}{c^{l}-a^{m}},\;c^{l}<x\leq a^{m}\\ \frac{a^{m}-x}{a^{m}-c^{u}},\;a^{m}\leq x<c^{u}\\ 1,\;x\geq c^{u} \end{array}\right.$$

**Figure 4:**
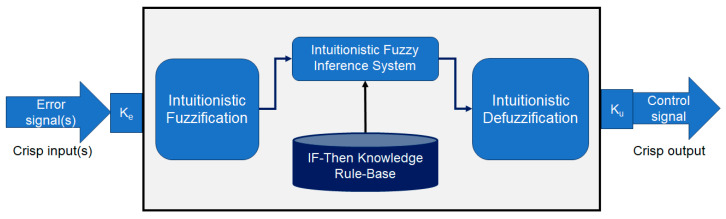
The basic workflow of intuitionistic fuzzy controller.

The basic workflow of an intuitionistic fuzzy logic controller (IFLC) is shown in Figure 4. The error signal, which computes as a crisp input value the difference between the desired heartbeats and the actual sensor output, is the input of the IFLC. Typically, the IFLC produces a real output value that provides the necessary control signal. Intuitionistic fuzzification, an intuitionistic fuzzy inference system (FIS) with an if–then rule base, and intuitionistic defuzzification are the main elements of IFLC [24,38]. The error signal is scaled by gain *K_e_* in the intuitionistic fuzzification stage and then allocated to each IFS in a given input universe of discourse *x*∈ℝ with a membership function δ and non-membership function β of the interval [0, 1]. The knowledge rule base of the inference engine is constructed by a set of *i^th^* intuitionistic if–then rules in (18).

(17) $$R_{i}^{\delta }:if\;x_{1}\;is\;A_{1,i}^{\delta }\;AND\;x_{2}\;is\;A_{2,i}^{\delta }\;AND\;\cdots AND\;x_{n}\;is\;A_{n,i}^{\delta }\;THEN\;z_{1}^{\delta }\;is\;B_{1,i}^{\delta }\;AND\;\cdots \;z_{m}^{\delta }\;is\;B_{m,i}^{\delta }\;\;\;R_{i}^{\beta }:if\;x_{1}\;is\;A_{1,i}^{\beta }\;AND\;x_{2}\;is\;A_{2,i}^{\beta }\;AND\;\cdots AND\;x_{n}\;is\;A_{n,i}^{\beta }\;THEN\;z_{1}^{\beta }\;is\;B_{1,i}^{\beta }\;AND\;\cdots \;z_{m}^{\beta }\;is\;B_{m,i}^{\beta }$$

For all *M* fuzzy rules *R_i_*, *i* = 1, 2, …, *m*, FIS^δ^ and FIS^β^ give the resulted outputs *z^δ^* and *z^β^* based on the membership function *δ_B_* and non-membership function *β_B_*, respectively, using the Center-of-Gravity (COG) defuzzification step [26,38]. In the end, the overall crisp output or the control signal *u* of the IFLC in (19) presents a linear collection of *z^δ^* and *z^β^* with the uncertainty value *σ_c_*, where 0 ≤ *σ_c_* ≤ 1. The scaling output gain *K_u_* is carefully selected to achieve the desired system response.

(18) $$u_{c}=K_{u}\left(\left(1-\sigma_{c}\right)z^{\delta }+\sigma_{c}z^{\beta }\right)$$

### 2.3. Developed Controller of Dual-Sensor Pacemakers

**Figure 5:**
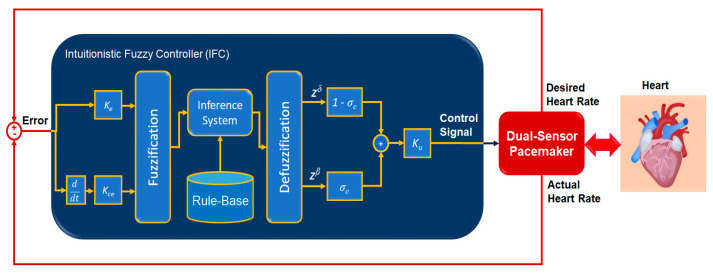
Workflow of developed intuitionistic fuzzy control for implanted dual-sensor pacemakers.

**Figure 6:**
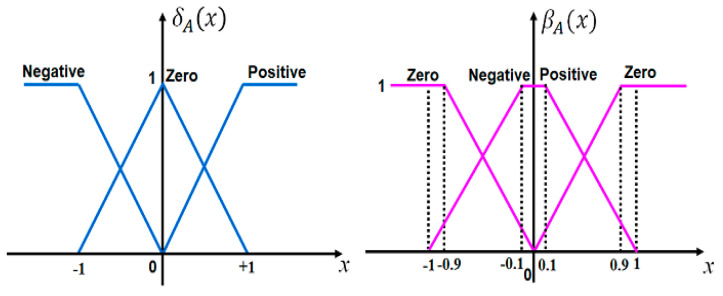
Membership functions (*δ*) and non–membership functions (*β*) for input–output fuzzification and defuzzification steps of the developed pacemaker control system.

Figure 5 presents the workflow of the developed IFC to regulate dual-sensor pacemakers. Two inputs of the IFC are the error and the change in error signals, which are scaled by the gains, *Ke* and *Kce*, respectively. The error signal represents the difference between the actual heart rate and the desired value of heart rates according to the body status. The control signal of the developed IFC is the duration of the pulsed heart rate to be generated by the cardiac pacemaker. Figure 6 depicts the triangular IFS functions of both fuzzification and defuzzification steps. They include three subsets of linguistic terms, which are Negative (N), Zero (Z), and Positive (P) on the normalized input and output ranges from −1 to +1. We assumed the uncertainty or hesitancy value is 0.0001 as given in (7). To generate intuitionistic fuzzy output, the if–then rule base of the intuitionistic fuzzy engine is designed as illustrated in Table 1.

## 3. Results and Evaluation

All simulation tests of the developed IFLC of the dual-sensor pacemaker have been implemented and executed by using the MATLAB/Simulink^®^ R2023b software and Fuzzy Logic Toolbox^TM^ V23.2. The parameters of the developed IFLC were manually adjusted to ensure reliable performance during all tested cases of dual-sensor pacemakers. These parameters are scaling input-output gains, *Ke* = 0.75, *Kce* = 0.75, and *Ku* = 100.0, as shown in Figure 5. The hesitancy value *σ_c_* is 0.0001. 

Table 2 illustrates all targeted or preset heart rates of six patients during three daily body activities, i.e., at rest, walking, and jogging [13,30,39,40]. The tested data of cardiac patients include six cases of three females and three males between 45 and 66 years old. Average preset heart rates are 86.2 ± 5.17 bpm at rest, 96.8 ± 4.8 bpm for walking, and 112.2 ± 4.5 bpm for jogging. The minimum preset heart rate is defined for case 6, as 80 ± 5 bpm at the rest activity, while the maximum preset heart rate is 122 ± 5 bpm during the jogging case 4, as given in Table 2. 

**Figure 7:**
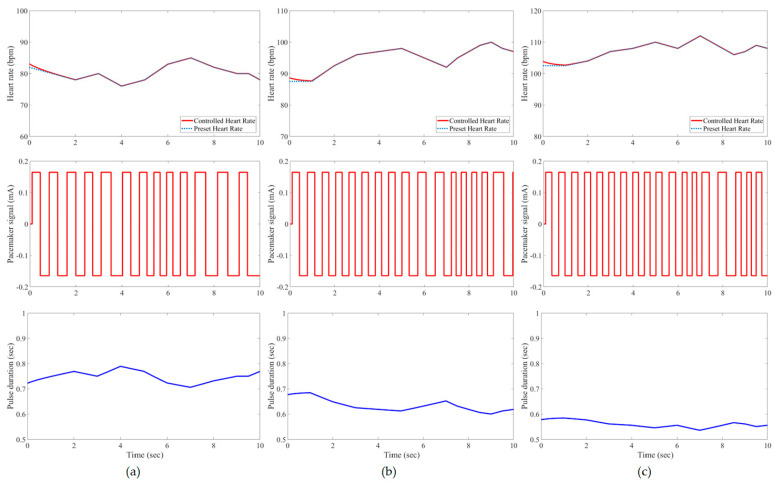
Results of the tested case 1 using developed pacemaker control: (**a**) at rest; (**b**) walking; and (**c**) jogging. The pulses of the pacemaker are shown in the second row, and the control signal is shown in the third row.

**Figure 8:**
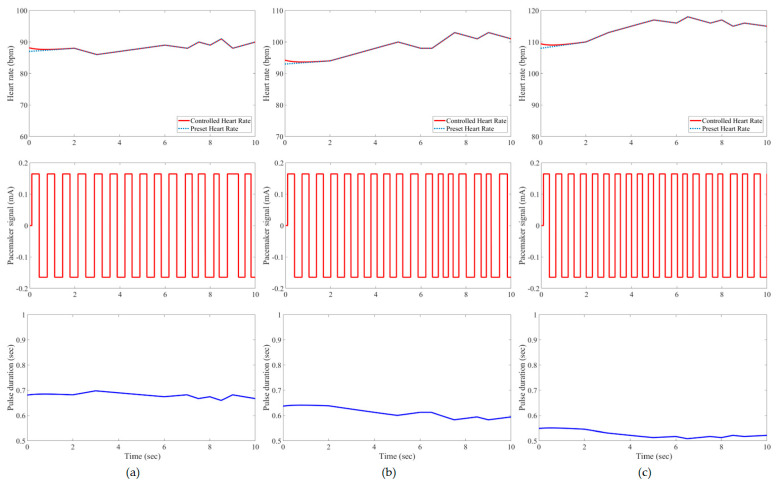
Results of the tested case 2 using developed pacemaker control: (**a**) at rest; (**b**) walking; and (**c**) jogging.

**Figure 9:**
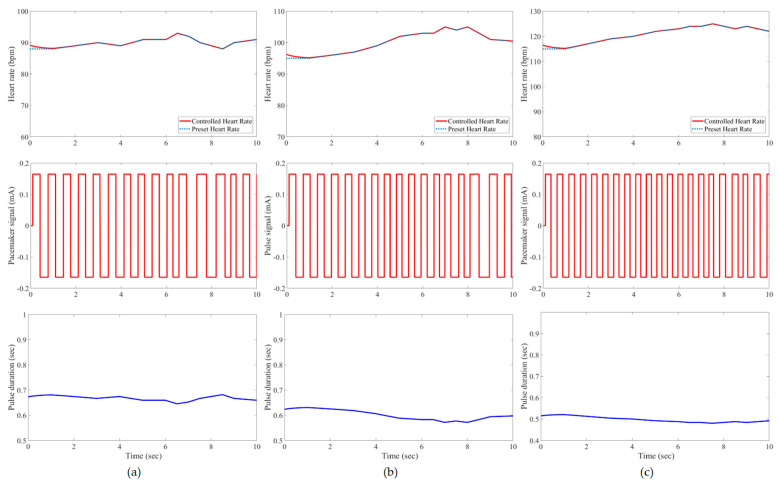
Results of the tested case 3 using developed pacemaker control: (**a**) at rest; (**b**) walking; and (**c**) jogging.

**Figure 10:**
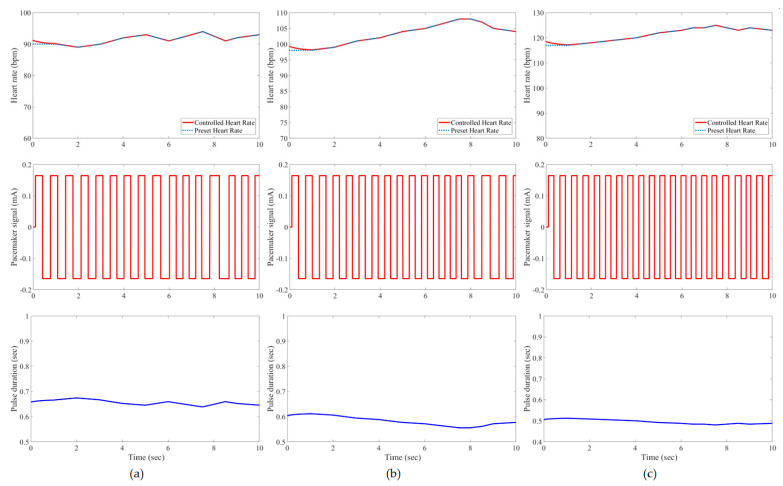
Results of the tested case 4 using developed pacemaker control: (**a**) at rest; (**b**) walking; and (**c**) jogging.

**Figure 11:**
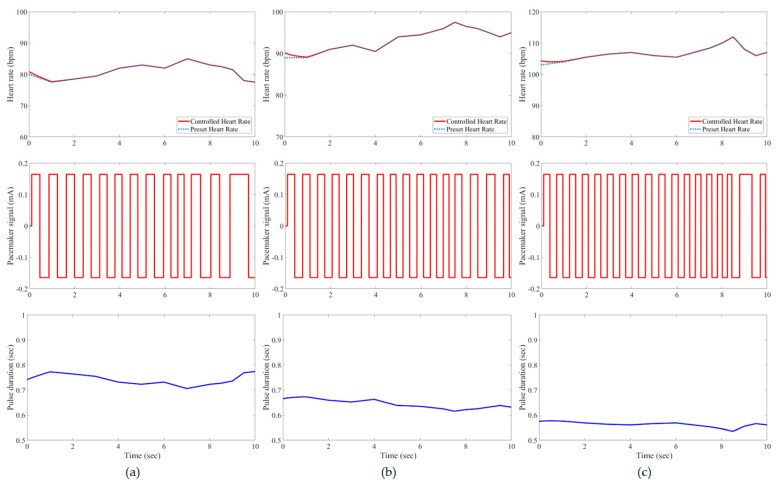
Results of the tested case 5 using developed pacemaker control: (**a**) at rest; (**b**) walking; and (**c**) jogging.

**Figure 12:**
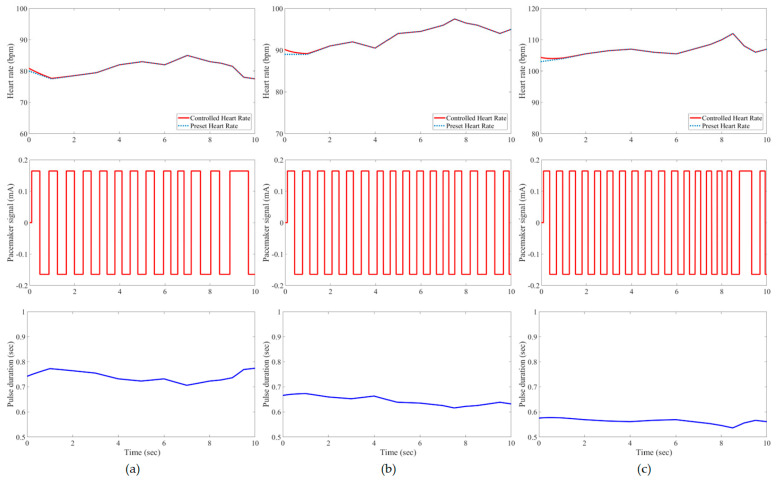
Results of the tested case 6 using developed pacemaker control: (**a**) at rest; (**b**) walking; and (**c**) jogging.

In this study, all pacemaker pulses have a constant amplitude of ±0.165 mA, but the corresponding duration is variable to represent the control action of the developed IFLC. The control signal to a pacemaker typically has a duration that is inversely proportionate to the target heart rates. Figure 7, Figure 8, Figure 9, Figure 10, Figure 11 and Figure 12 depict the tracking results of six tested patients’ preset heart rates during three daily activities—walking, running, and resting—using the developed IFLC of the implanted pacemaker. In Figure 7, the controlled pulse duration of the dual-sensor pacemaker for case 1 is 0.53 to 0.79 s to manage the heart rates of 113 to 76 bpm during three body activities. The preset heart rates of case 4 are the highest values among all the tested cases, as listed in Table 2. Therefore, the minimum pulse durations of the controlled pacemaker are produced, which are 0.65, 0.59, and 0.49 s for the body activity status at rest (92 bpm), walking (102 bpm), and jogging (122 bpm), respectively, as depicted in Figure 10. The developed controller of the implanted dual-sensor pacemaker showed a successful pacing-rate response for all tested cases to achieve the longest pulse duration of 0.79 s at the rest states of cases 1 and 6 (see Figure 7 and Figure 12), while the shortest pulse duration of 0.49 s is produced for cases 3 and 4 at the jogging states, as shown in Figure 9 and Figure 10. That allows the heart to provide the patient’s body with sufficient blood amount according to the daily activity.

To emphasize the benefits and accurate performance of our developed controller, Table 3 compares the developed IFLC against other fuzzy and neural network controllers in previous studies [15,39,40] utilizing the same pacemaker model and the preset heart rates of all tested cases. The performance results of the RBF neural network controller [15] are not available for cases 5 and 6, but other data results demonstrate the general behavior of adaptive RBF networks for controlling dual-sensor pacemakers. The developed IFLC achieved the smallest values of the root mean squared error (RMSE) in the range of 0.17 for case 6 at the rest state to 0.26 for case 4 at the jogging state; see Table 3. The RBF neural network control achieved better maximum error values than the developed controller for most cases at the rest and the jogging states. However, the developed IFLC is still capable of achieving the lowest values of the maximum errors for tracking the preset heart rates in almost all walking cases less than 1.85%, as illustrated in Table 3. 

## 4. Discussion

Automatic closed-loop control of dual-sensor pacemakers has been successfully achieved using our developed IFLC on six cardiac patients during three daily activities, i.e., at rest, walking, and jogging, as shown in Figure 7, Figure 8, Figure 9, Figure 10, Figure 11 and Figure 12. The developed IFLC demonstrated accurate results for tracked preset heart rates at any tested body condition. The implication of the non-membership functions with the uncertainty term *σ_c_*, as given in (17) and (18), plays a key role in the designed IFLC for improving controlled pacemakers over traditional fuzzy and fuzzy PID controllers in previous studies, as depicted in Figure 5 and Table 3. That allows an adaptive estimation of the pacemaker pulse duration, which is the control signal of the developed fuzzy controller in response to the body activity of patients.

The RMSE and maximum errors have been used as performance evaluation metrics of implanted pacemaker controllers [13,39]. In Table 3, the developed IFLC showed superior averaged performance over other types of fuzzy and RBF network controllers to regulate different pacing rates. The developed IFLC achieved significantly better performance than traditional fuzzy and fuzzy PID controllers by reducing the RMSE to 0.17 for case 6 and the maximum error of 0.49% for case 4, as presented in Table 3. Although the RBF network controller achieved lower maximum error values, e.g., 0.43% for case 3, than the developed controller, the IFLC achieved the best values of the RMSE for all cases. That means the steady-state performance of our developed IFLC is stable to give the best therapeutic results of the controlled dual-sensor pacemakers. Additionally, uncertainty handling is the main advantage of the designed IFLC against other competitive controllers in Table 3, as given in the hesitancy value in (7). Also, the developed IFLC can be considered as a generic representation of previous fuzzy controllers by adding non-membership function terms to achieve accurate control performance of the pacemaker, as depicted in Figure 6. Consequently, the computational cost of the designed IFLC is relatively higher than that of other classical fuzzy controllers, but the expected energy consumption for executing the IFLC algorithm is negligible to affect the lifespan of the current lithium batteries for implanted pacemakers. 

To further improve the pacing-rates regulation using our developed IFLC, auto-tuning parameters of the designed fuzzy controller can be accomplished using metaheuristic optimization techniques [24,41]. This optimization step could enhance the performance of the IFLC, minimizing the maximum errors of tracked preset heart rates to the lowest values. Also, merging neural networks with IFLC can constitute a good performance for time-varying nonlinear systems [42] similar to the dynamical behavior of cardiac pacemakers. However, the evaluated performance of the developed IFLC is still superior to other fuzzy and neural network controllers in previous studies for achieving the automated regulation of heart rates via implanted dual-sensor pacemakers as presented in Table 3. 

## 5. Conclusions

In this article, a novel IFLC has been successfully developed for controlling the electrical pulses of dual-sensor pacemakers. The developed controller can assist cardiac patients in achieving the preset heart rates with minimal RMSEs of 0.17 to 0.24 during three different daily activities: resting, walking, and jogging. The main parameters of the developed IFLC such as the input–output control gains and the hesitancy value are carefully tuned to achieve a desirable performance of tracked preset heart rates, as shown in Figure 7, Figure 8, Figure 9, Figure 10, Figure 11 and Figure 12. Moreover, the comparative evaluation of the developed IFLC versus other closed-loop controllers in the literature showed the outperformance of applied intuitionistic fuzzy for controlling therapeutic medical devices like implanted pacemakers.

The future work of this study includes adding more daily activities of the patients such as running and climbing stairs to cover all possible conditions of the heart. The future version of the designed IFLC can be improved to be suitable for single-sensor pacemakers and/or other types of cardiac pacemakers. Conducting preclinical studies will be arranged with collaborated cardiologists to implement and validate our developed IFLC of implanted dual-sensor pacemakers. 

## Figures and Tables

**Table 1:** Developed fuzzy rules for regulating dual-sensor pacemakers.

Change of Error (ce)	Error (e)		
	Negative	Zero	Positive
**Negative**	Negative	Negative	Zero
**Zero**	Negative	Zero	Positive
**Positive**	Zero	Positive	Positive

**Table 2:** Desired heart rates of six tested cases during daily body states.

Cardiac Patient (Sex, Age (Years))	Desired Heart Rates (bpm)		
	At Rest	Walking	Jogging
Case1 (Female, 66)	81 ± 5	94 ± 5	107 ± 5
Case 2 (Male, 54)	89 ± 5	98 ± 5	113 ± 5
Case 3 (Male, 48)	90 ± 5	100 ± 5	120 ± 5
Case 4 (Female, 45)	92 ± 5	103 ± 5	122 ± 5
Case 5 (Female, 58)	85 ± 6	92 ± 4	103 ± 3
Case 6 (Male, 62)	80 ± 5	94 ± 5	108 ± 4

**Table 3:** Comparative evaluation of the developed IFLC versus other closed-loop controllers in previous studies.

Control Method	Patient	At Rest		Walking		Jogging	
		RMSE	Maximum Error	RMSE	Maximum Error	RMSE	Maximum Error
Classical Fuzzy [39,40]	Case 1	2.38	4.88%	3.72	7.29%	6.40	8.26%
	Case 2	2.07	3.45%	4.24	6.34%	3.39	5.31%
	Case 3	3.14	5.38%	4.73	6.86%	2.76	4.35%
	Case 4	3.47	6.45%	3.36	4.08%	2.67	4.27%
	Case 5	1.81	2.63%	2.68	2.84%	2.68	5.01%
	Case 6	2.51	4.82%	2.27	3.91%	2.45	3.21%
Fuzzy PID [39,40]	Case 1	1.19	2.63%	1.14	2.27%	1.27	1.96%
	Case 2	0.91	2.30%	0.95	2.15%	1.23	2.77%
	Case 3	0.89	2.63%	0.87	2.08%	0.62	1.71%
	Case 4	1.09	2.13%	0.76	1.47%	0.64	1.71%
	Case 5	0.89	**1.72%**	1.35	2.27%	1.44	2.51%
	Case 6	0.82	1.92%	1.15	2.23%	0.66	**0.94%**
RBF Neural Network [15]	Case 1	0.64	1.68%	0.54	**0.57%**	0.90	**1.13%**
	Case 2	0.45	**1.22%**	0.68	**0.64%**	0.80	**0.54%**
	Case 3	0.49	**1.68%**	0.53	1.07%	0.71	**0.43%**
	Case 4	0.44	**0.78%**	0.51	0.99%	0.74	**0.56%**
	Case 5	–	–	–	–	–	–
	Case 6	–	–	–	–	–	–
Developed IFLC	Case 1	0.21 *	**1.62%**	**0.21**	1.85%	**0.23**	2.21%
	Case 2	**0.21**	1.60%	**0.21**	1.41%	**0.25**	1.55%
	Case 3	**0.20**	1.76%	**0.22**	**0.78%**	**0.25**	1.00%
	Case 4	**0.21**	1.23%	**0.23**	**0.49%**	**0.26**	1.00%
	Case 5	**0.20**	2.35%	**0.21**	**1.60%**	**0.23**	**2.12%**
	Case 6	**0.17**	**1.58%**	**0.22**	**1.64%**	**0.24**	2.38%

* Bold values indicate the best performance result of the controlled pacemaker.

## Data Availability

The data that support the findings of this research are publicly available, as indicated in the references.
